# The Ionomic Study of Vegetable Crops

**DOI:** 10.1371/journal.pone.0160273

**Published:** 2016-08-01

**Authors:** Toshihiro Watanabe, Eriko Maejima, Tomoko Yoshimura, Masaru Urayama, Aiko Yamauchi, Masako Owadano, Ryosuke Okada, Mitsuru Osaki, Yoshinori Kanayama, Takuro Shinano

**Affiliations:** 1 Research Faculty of Agriculture, Hokkaido University, Kita-9, Nishi-9, Kitaku, Sapporo, 0608589, Japan; 2 Graduate School of Agricultural Science, Tohoku University, Sendai, 9818555, Japan; 3 Agricultural Radiation Research Center, NARO Tohoku Agricultural Research Center, 50 Aza Harajyukuminami, Arai, Fukushima, 9602156, Japan; United States Department of Agriculture, Agricultural Research Service, UNITED STATES

## Abstract

Soil contains various essential and nonessential elements, all of which can be absorbed by plants. Plant ionomics is the study of the accumulation of these elements (the ionome) in plants. The ionomic profile of a plant is affected by various factors, including species, variety, organ, and environment. In this study, we cultivated various vegetable crop species and cultivars under the same field conditions and analyzed the level of accumulation of each element in the edible and nonedible parts using ionomic techniques. The concentration of each element in the edible parts differed between species, which could be partly explained by differences in the types of edible organs (root, leaf, seed, and fruit). For example, the calcium concentration was lower in seeds and fruit than in other organs because of the higher dependency of calcium accumulation on xylem transfer. The concentration of several essential microelements and nonessential elements in the edible parts also varied greatly between cultivars of the same species, knowledge of which will help in the breeding of vegetables that are biofortified or contain lower concentrations of toxic elements. Comparison of the ionomes of the fruit and leaves of tomato (*Solanum lycopersicum*) and eggplant (*S*. *melongena*) indicated that cadmium and boron had higher levels of accumulation in eggplant fruit, likely because of their effective transport in the phloem. We also found that homologous elements that have been reported to share the same uptake/transport system often showed significant correlation only in a few families and that the slopes of these relationships differed between families. Therefore, these differences in the characteristics of mineral accumulation are likely to affect the ionomic profiles of different families.

## Introduction

At least 17 elements are known to be essential nutrients for plants; however, plants also absorb and accumulate various nonessential elements [1, 2]. In contrast, humans need at least 25 elements [3], a major source of which is crops. Mineral malnutrition is a widespread public health issue in both developing and developed countries, with up to two-thirds of the world’s population being considered at risk of inadequate intakes of one or more essential mineral elements [3]. Iron (Fe) and zinc (Zn) deficiencies are common in humans, particularly in developing countries [4]. One possible way of addressing this issue is through biofortification, whereby crops are developed that contain essential mineral elements, increasing their nutritional value for humans [5].

Some crops also contain toxic elements, which can represent a public health threat. Crops that are cultivated on arable land that is polluted with heavy metals and metalloids often contain a high concentration of these elements in their edible parts. In some Asian countries, for example, cadmium (Cd) contamination in rice (*Oryza sativa*) grains poses a serious threat to public health; therefore, many studies have been conducted to address this issue [6, 7]. In addition, crops grown in soil that contains radioactive elements such as cesium (Cs) or strontium (Sr) as a result of a nuclear accident can pose a risk to human health. Therefore, comprehensive information about the potential for different species and cultivars of crops to accumulate each type of element in its edible parts is very important for human health.

Ionomics is the study of the accumulation of all metals, metalloids, and nonmetals in living organisms using high-throughput elemental analysis technologies (e.g. Inductively Coupled Plasma-Mass Spectrometry (ICP-MS) and ICP-Atomic Emission Spectrometry (ICP-AES)) [8] and can be applied to various types of plant science studies. Ionomics has the advantage in revealing network among various mineral elements in organism. For example, ionomic analyses have been conducted to isolate the genes that are responsible for mineral transport and homeostasis in plants [9, 10]. Furthermore, ionomics can also be applied to investigate phylogenetic and environmental influences on plant mineral accumulation [11–13]. Thus, ionomics is a powerful tool to study mineral dynamics in plants. Recently, ionomics have also been applied to seeds of staple crops, such as cereals and beans, and are expected to contribute to improving the malnutrition and food safety [14, 15]. However, there are very few reports of application of this technology to the edible parts of non-staple crops, particularly in vegetable crops.

In the present study, therefore, we used ionomics to comprehensively characterize mineral accumulation in the edible parts of various crop species. We cultivated a number of different vegetable crop species, including various cultivars, under the same field conditions and analyzed the ionome of their edible parts. Moreover, we also analyzed the ionome in nonedible leaves and compared it with that in edible fruits to understand the ionome distribution between source and sink organs. The findings of this study will be useful for enhancing the nutritional value and safety of these crops in the future.

## Materials and Methods

### Cultivation

In 2011, 24 vegetable crop species, including two rootstocks, were cultivated in the experimental field of Hokkaido University, Japan. The crop species that were used in this study are shown in Table 1. The experimental field had a Gleyic Fluvisol soil type, the general chemical properties (exchangeable elements, Truog P, and soil pH) of which before cultivation are described in S1 Table. The soil analysis was conducted by the method of Watanabe et al. [2]. For all crop species, nitrogen (N), phosphorus (P), and potassium (K) fertilizers were uniformly applied as ammonium sulfate (140 kg N ha^−1^), superphosphate (130 kg P_2_O_5_ ha^−1^), and potassium sulfate (100 kg K_2_O ha^−1^). Seeds of carrot (*Daucus carota* subsp. *sativus*), radish (*Raphanus sativus* var. *longipinnatus*), turnip (*Brassica rapa* var. *rapa*), komatsuna (*B*. *rapa* var. *perviridis*), bok-choy (*B*. *rapa* var. *chinensis*), nabana (*B*. *rapa* var. *nippo-oleifera*), napa cabbage (*B*. *rapa* var. *pekinensis*), garland chrysanthemum (*Glebionis coronaria*), podded pea (*Pisum sativum*), green pea (*P*. *sativum*), and kidney bean (*Phaseolus vulgaris*) were directly sown in the field. Seeds of the remaining species were sown in the 300 mL pot containing soils, grown in a greenhouse at Hokkaido University, and transplanted to the field. The sowing and transplanting details are provided in Table 1. All species used in this study are usually cultivated in this prefecture at this season.

**Table 1:** List of the vegetable species used in this study and details about the sowing, transplanting, and sampling procedures.

Species number	Family	Common name^a^	Scientific name	Major edible part	Analyzed part	Intrarow × row(cm)	Sowing date	Transplanting date	Sampling date	No. samples
1	Malvaceae	Okra (2)	*Abelmoschus esculentus* (L.) Moench	Fruit	Fruit	50 × 60	May-5	Jun-17	Aug-10	1 plant/replicate
2	Apiaceae	Carrot (6)	*Daucus carota* subsp. *sativus*(Hoffm.) Arcang.	Root	Root	13 × 13	May-24	-	Sep-13	1 plant/replicate
3	Brassicaceae	Radish (17)	*Raphanus sativus* L. var. *longipinnatus* L.H.Bailey	Root	Root	30 × 90	May-24	-	Jul-11	1 plant/replicate
4	Brassicaceae	Turnip (14)	*Brassica rapa* L. var. *rapa*	Root	Root	30 × 90	May-24	-	Jul-7	1 plant/replicate
5	Brassicaceae	Broccoli (10)	*Brassica oleracea* L. var. *italica*	Flower bud	Flower bud	50 × 90	Apr-26	Jun-9	13-Jul or 4-Aug	1 plant/replicate
6	Brassicaceae	Cauliflower (3)	*Brassica oleracea* L. var. *botrytis*	Flower bud	Flower bud	50 × 90	Apr-26	Jun-9	Jul-19	1 plant/replicate
7	Brassicaceae	Cabbage (18)	*Brassica oleracea* L. var. *capitata*	Leaf	Leaf	50 × 90	Apr-26	May-25	Jul-26	1 plant/replicate
8	Brassicaceae	Komatsuna (8)	*Brassica rapa* L. var. *perviridis*	Leaf	Leaf	13 × 13	May-24	-	Jun-27	2 plants/replicate
9	Brassicaceae	Bok-choy (7)	*Brassica rapa* L. var. *chinensis*	Leaf	Leaf	13 × 13	May-24	-	Jul-6	2 plants/replicate
10	Brassicaceae	Nabana (2)	*Brassica rapa* L. var. *nippo-oleifera*	Leaf	Leaf	13 × 13	May-24	-	Jun-27	2 plants/replicate
11	Brassicaceae	Napa cabbage (14)	*Brassica rapa* L. var. *pekinensis* Rupr.	Leaf	Leaf	50 × 90	May-26	-	Aug-4	1 plant/replicate
12	Asteraceae	Garland chrysanthemum (2)	*Glebionis coronaria* (L.) Cass. ex Spach	Leaf	Leaf	13 × 13	May-24	-	Jul-11	2 plants/replicate
13	Asteraceae	Lettuce (24)	*Lactuca sativa* L.	Leaf	Leaf	50 × 90	Apr-20	May-25	Jul-6	1 plant/replicate
14	Cucurbitaceae	Cucumber (14)	*Cucumis sativus* L.	Fruit	Fruit	50 × 60	Apr-27	Jun-7	Jul-19	2 fruits from 2 plants/replicate
15	Solanaceae	Tomato (16)	*Solanum lycopersicum* L.	Fruit	Fruit, Leaf	50 × 90	Apr-20	Jun-8	Aug-17	1 fruit from 1 plant/replicate, 5 fully expanded leaves from 1 plant/replicate
16	Solanaceae	Grape tomato (2)	*Solanum lycopersicum* L.	Fruit	Fruit, Leaf	50 × 90	Apr-20	Jun-8	Aug-17	5 fruits from 1 plant/replicate, 5 fully expanded leaves from 1 plant/replicate
17	Solanaceae	Tomato rootstock (10)	*Solanum lycopersicum* L.	Fruit	Fruit, Leaf	50 × 90	Apr-20	Jun-8	Aug-17	3 fruits from 1 plant/replicate, 5 fully expanded leaves from 1 plant/replicate
18	Solanaceae	Eggplant (7)	*Solanum melongena* L.	Fruit	Fruit, Leaf	50 × 90	Apr-20	Jun-22	Jul-25	1 fruit from 1 plant/replicate, 2 fully expanded leaves from 1 plant/replicate
19	Solanaceae	Eggplant rootstock (5)	*Solanum melongena* L., *S*. *torvum* Sw., *S*. *integrifolium* Poir.	-	Leaf	50 × 90	Apr-20	Jun-22	Jul-25	2 fully expanded leaves from 1 plant/replicate
20	Fabaceae	Podded pea (6)	*Pisum sativum* L.	Pod	Pod with seed	30 × 90	Jun-4	-	Jul-28	10 pods from 3 plants/replicate
21	Fabaceae	Green pea (3)	*Pisum sativum* L.	Seed	Seed, Pod with seed	30 × 90	Jun-4	-	Aug-8	10 pods from 3 plants/replicate
22	Fabaceae	Kidney bean (8)	*Phaseolus vulgaris* L.	Pod	Pod with seed	30 × 90	Jun-4	-	Jul-28	10 pods from 3 plants/replicate
23	Amaryllidaceae	Chinese chive (6)	*Allium tuberosum* Rottler ex Spreng.	Leaf	Leaf	50 × 90	Apr-20	Jun-29	Oct-14	1 plant/replicate
24	Amaryllidaceae	Green onion (25)	*Allium fistulosum* L.	Leaf	Leaf	5 × 100	Apr-20	Jun-22	Oct-14	1 plant/replicate

^a^ The number of cultivars is indicated in parentheses. The cultivar names are not included in this paper to avoid any conflicts of interest.

Each cultivar was cultivated in at least four rows with sufficient row length. The edible part of each cultivar was randomly collected avoiding the borders of each cultivation plot, with three replicates. In addition, both the fruit and the leaves were sampled from tomato (*Solanum lycopersicum*) (both grape tomato and rootstock) and eggplant (*S*. *melongena*), whereas only the leaves were sampled from the rootstock of eggplant. Leaf sampling involved collecting the largest fully expanded leaf from each plant. The sampling details are provided in Table 1. Sampling for crops harvested multiple times, such as cucumber, tomato, eggplant, and beans, was conducted at the mid-harvest stage. All samples were dried in an oven at 70°C for 7 days, following which they were weighed and then ground for mineral analysis.

### Mineral analysis

Plant samples were digested in 2 mL of 61% (w/v) HNO_3_ (EL grade; Kanto Chemical, Tokyo, Japan) at 110°C in a DigiPREP apparatus (SCP Science, QC, Canada) for approximately 2 h until the solution had almost disappeared. When the samples had cooled, 0.5 mL H_2_O_2_ (semiconductor grade; Santoku Chemical, Tokyo, Japan) was added and the samples were heated at 110°C for a further 20 min. Once digestion was complete, the tubes were cooled and made up to a volume of 10 mL by adding 2% (w/v) HNO_3_ in Milli-Q water. The concentrations of the following elements were measured using an ICP-MS (ELAN DRC-e; Perkin Elmer, Waltham, MA, USA): lithium (Li), boron (B), sodium (Na), K, chromium (Cr), cobalt (Co), nickel (Ni), arsenic (As), molybdenum (Mo), Cd, magnesium (Mg), aluminum (Al), P, calcium (Ca), manganese (Mn), Fe, copper (Cu), Zn, Sr, barium (Ba), and Cs.

### Statistical analyses

All statistical analyses were performed using Sigmaplot 11.0 (Systat Software, Inc., San Jose, CA, USA) and Minitab 14 (Minitab Inc., State College, PA, USA).

## Results and Discussion

### Ionome of the edible parts

**Fig 1:**
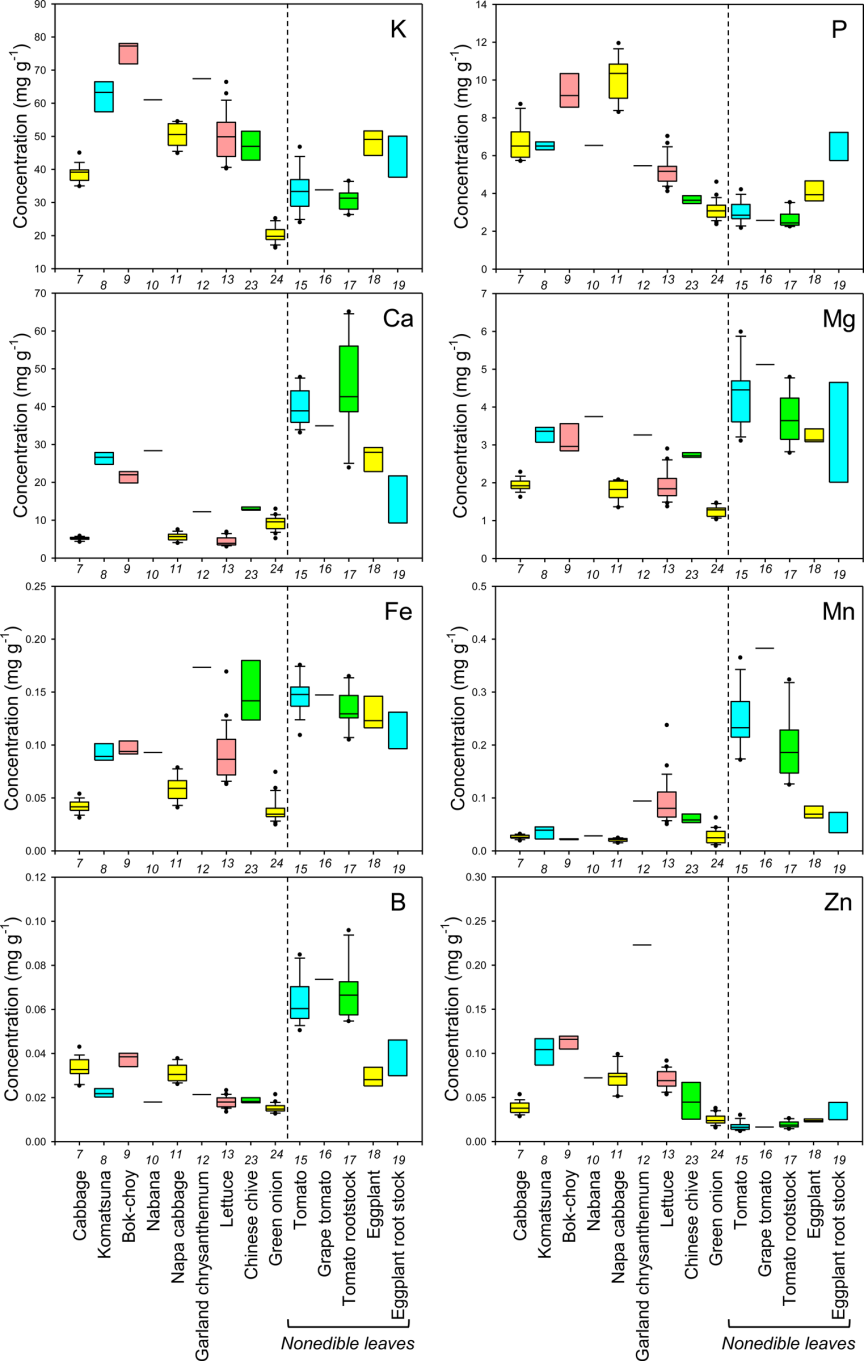
Boxplots showing the concentration of K, P, Ca, Mg, Fe, Mn, B, and Zn in leaves of various crop species. Concentrations in nonedible leaves are also shown. Numbers in x-axis indicate the species number in Table 1.

**Fig 2:**
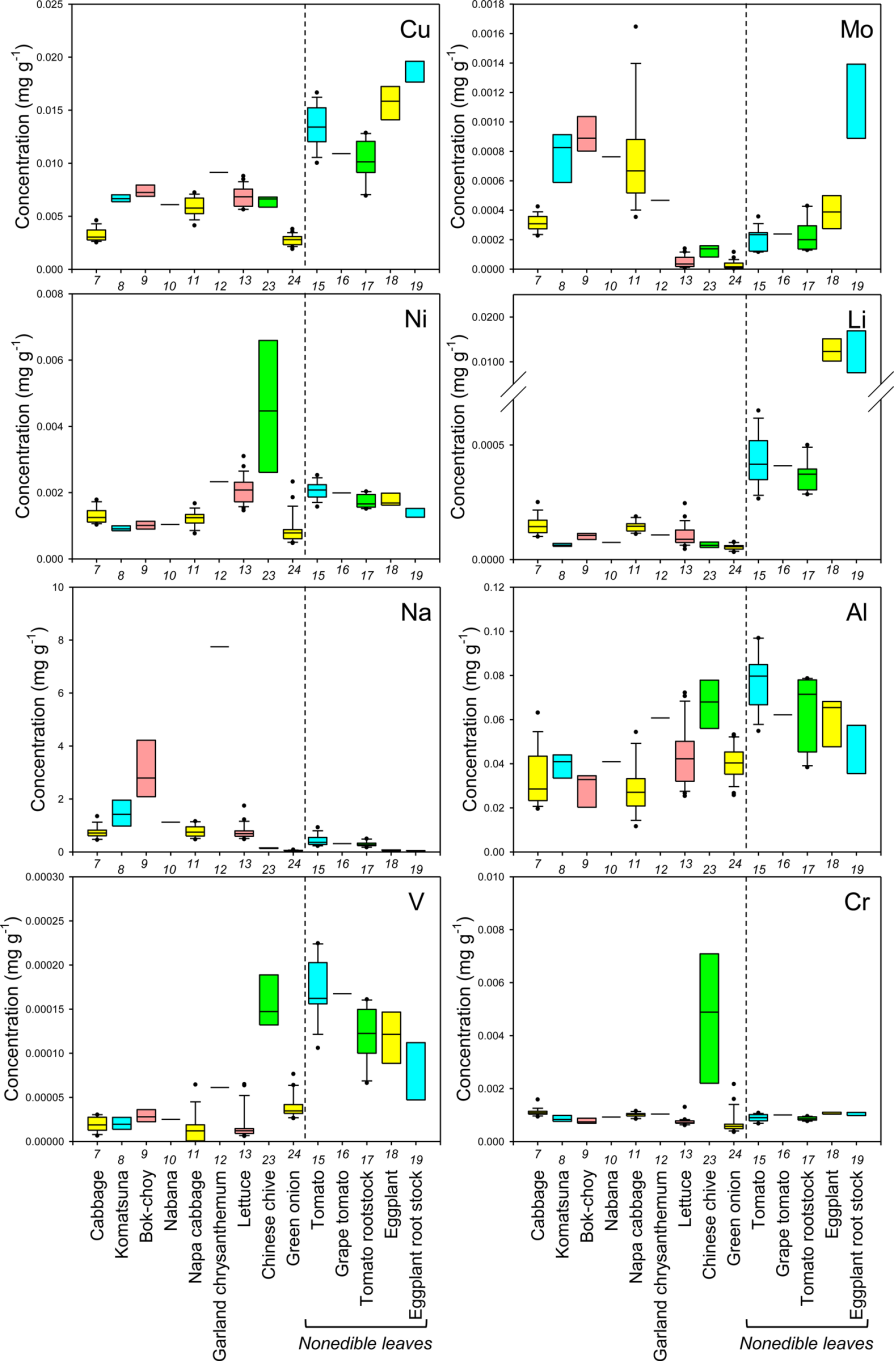
Boxplots showing the concentration of Cu, Mo, Ni, Li, Na, Al, V, and Cr in leaves of various crop species. Concentrations in nonedible leaves are also shown. Numbers in x-axis indicate the species number in Table 1.

**Fig 3:**
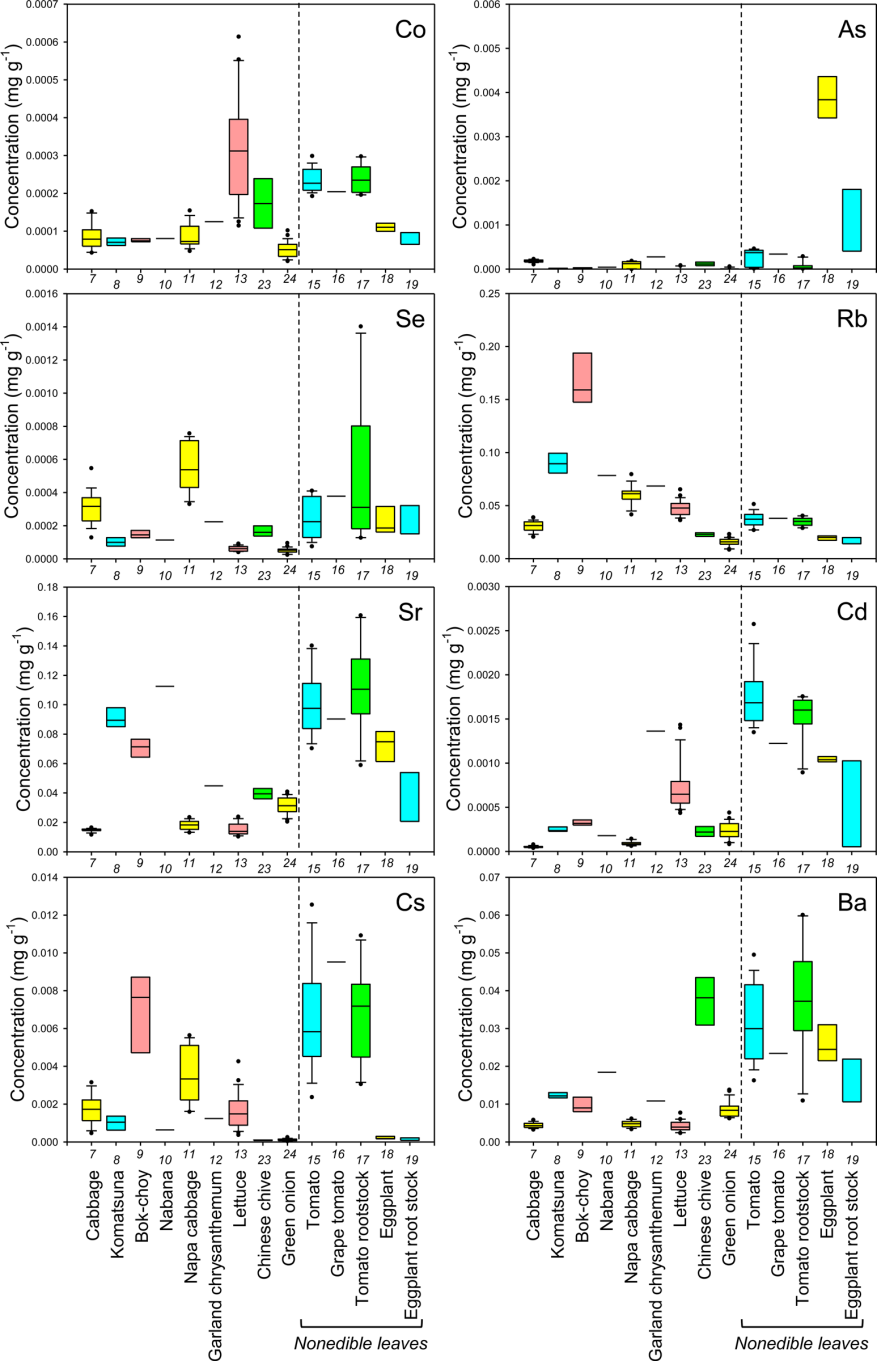
Boxplots showing the concentration of Co, As, Se, Rb, Sr, Cd, Cs, and Ba in leaves of various crop species. Concentrations in nonedible leaves are also shown. Numbers in x-axis indicate the species number in Table 1.

**Fig 4:**
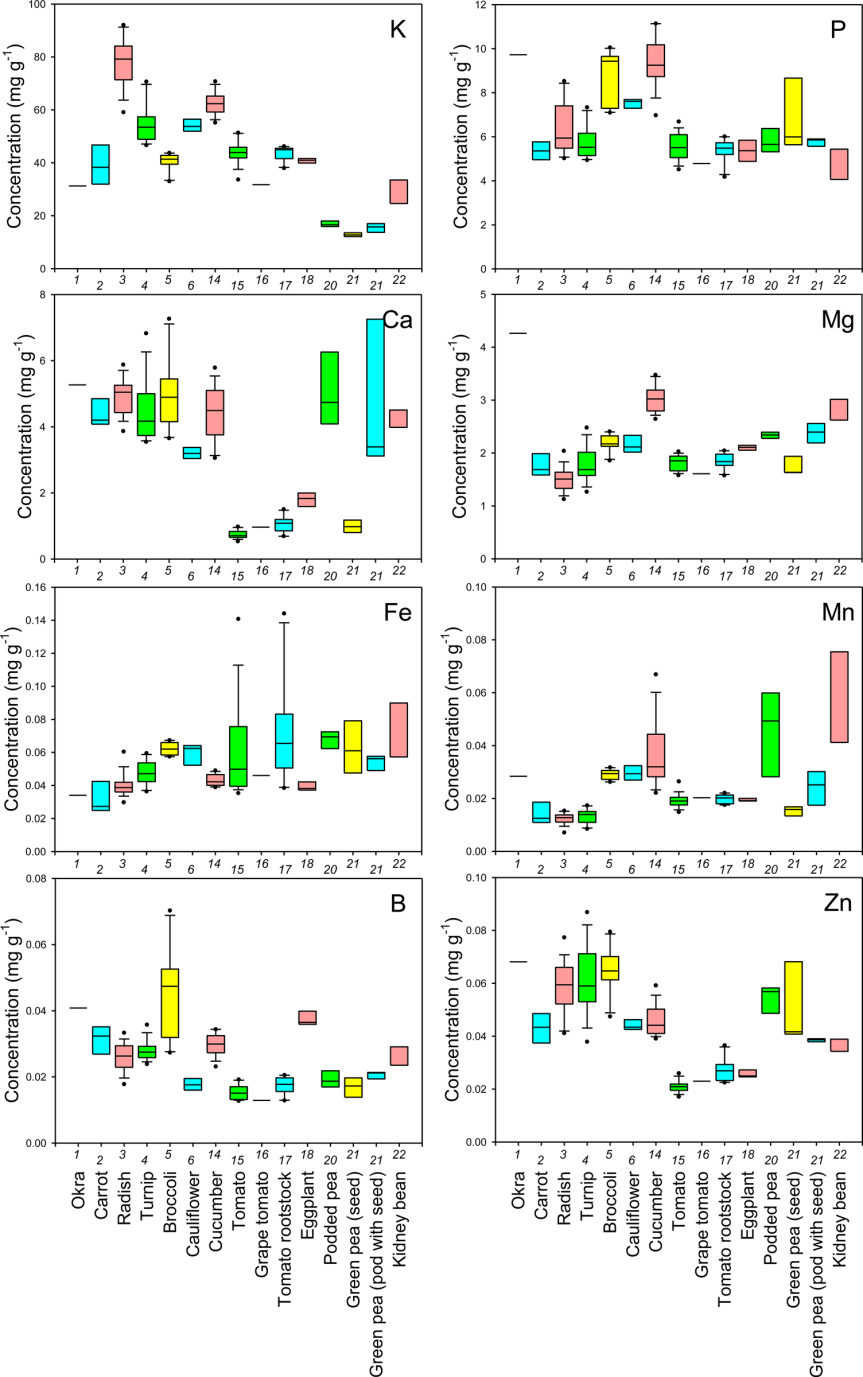
Boxplots showing the concentration of K, P, Ca, Mg, Fe, Mn, B, and Zn in non-leaf edible parts of various crop species. Numbers in x-axis indicate the species number in Table 1.

**Fig 5:**
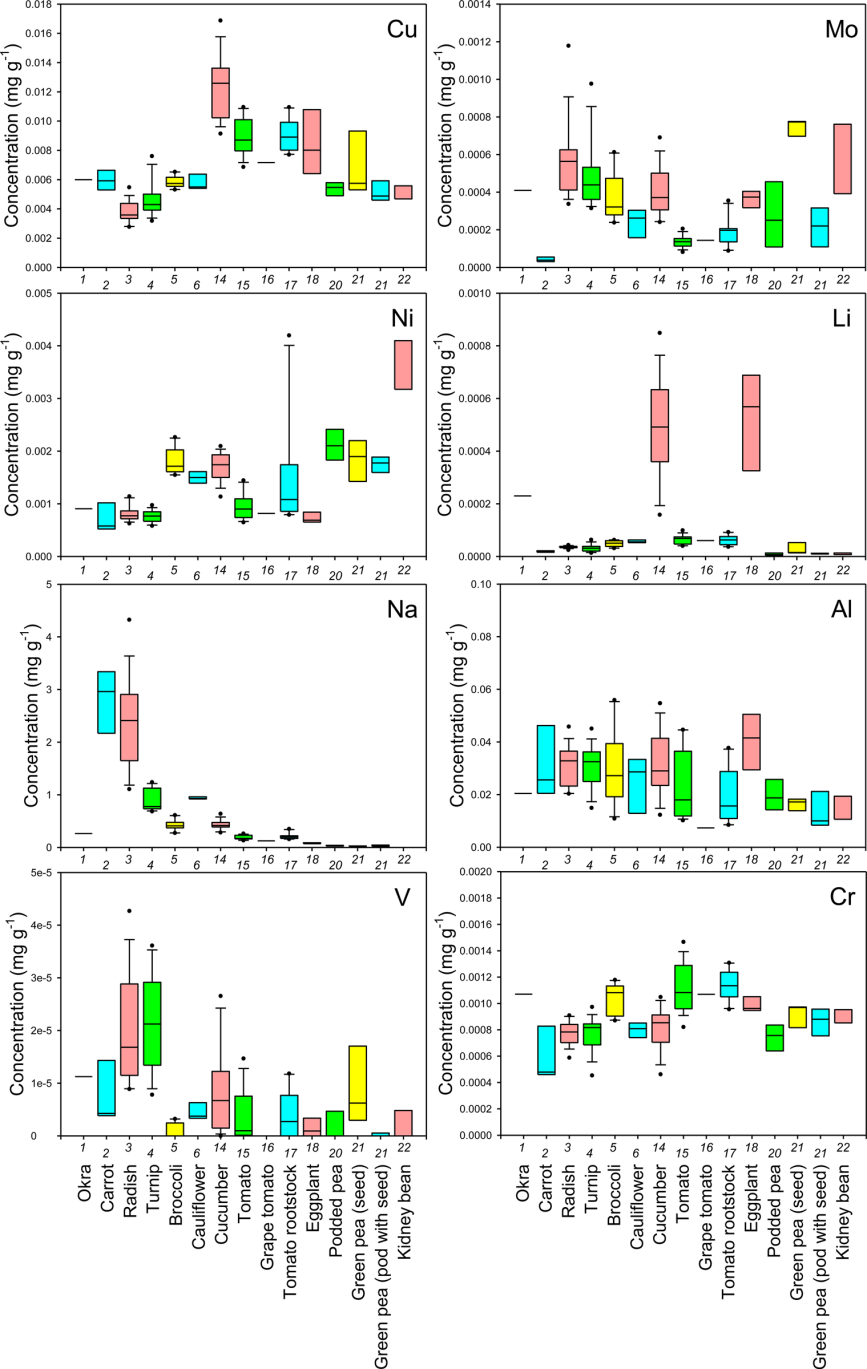
Boxplots showing the concentration of Cu, Mo, Ni, Li, Na, Al, V, and Cr in non-leaf edible parts of various crop species. Numbers in x-axis indicate the species number in Table 1.

**Fig 6:**
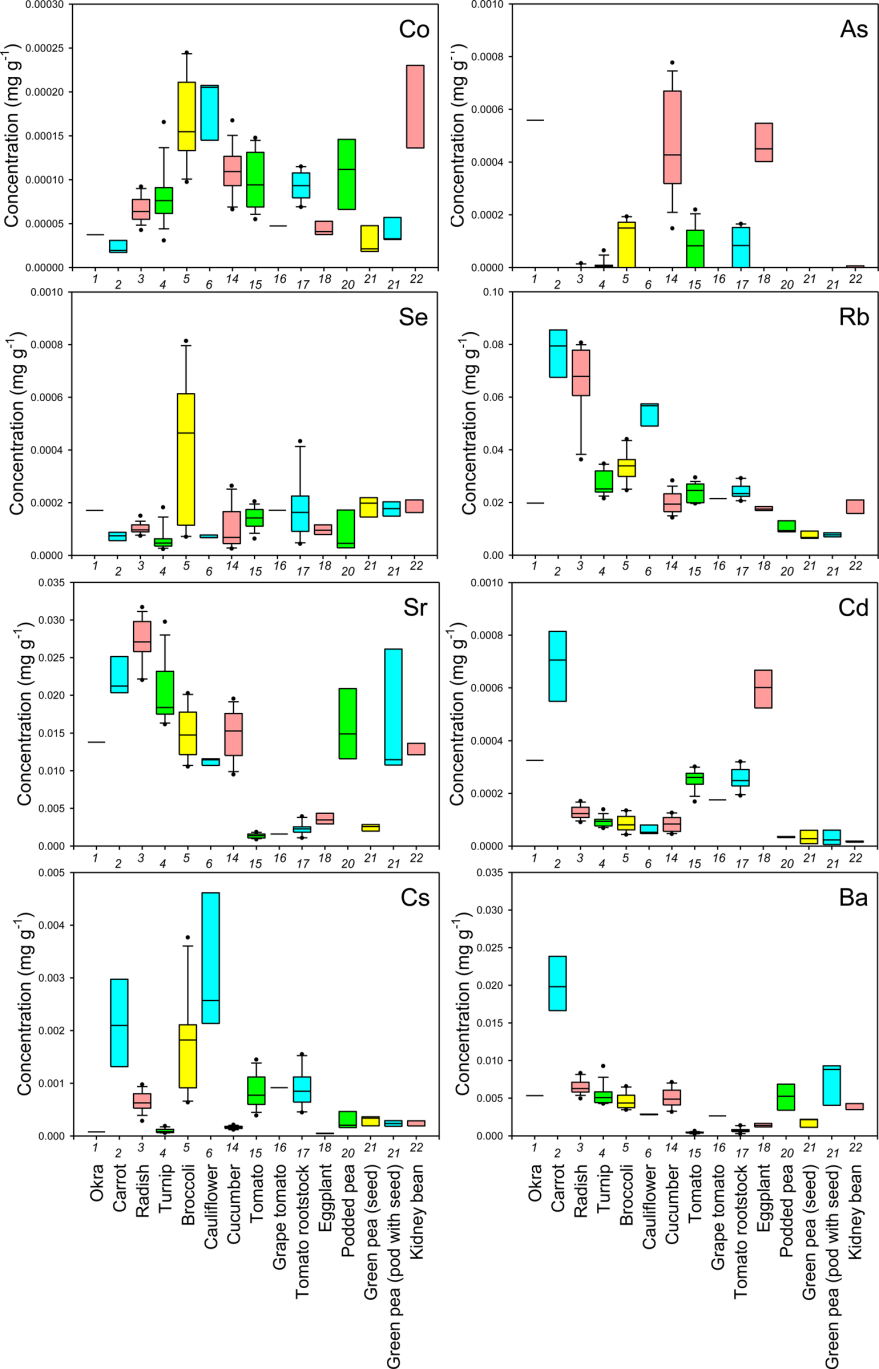
Boxplots showing the concentration of Co, As, Se, Rb, Sr, Cd, Cs, and Ba in non-leaf edible parts of various crop species. Numbers in x-axis indicate the species number in Table 1.

First, to examine the reliability of cultivation and sampling methods of this study, cultivar Komatsuna4 was cultivated in 2012 at a different site in the same field under the same cultivation conditions as in 2011. The results showed that the mineral concentrations in leaves of Komatsuna4 in 2012 were almost the same as those in 2011 (S2 Table), implying that the methods in this study are reliable. The concentrations of each mineral in the edible parts of the sampled crops were shown as boxplots in Figs 1–6 and all data were shown in S3 Table. The concentrations in the nonedible leaves were also shown for comparison (Figs 1–3). A boxplot is a simple and convenient graphical tool that is widely used to visualize the distribution of continuous univariate data [16]. Both interspecific and intraspecific variations in the concentrations of elements in edible parts were observed though the nature of the edible parts differed between crop species (Figs 1–6). In edible parts, the concentration of K was lower in both the seeds and the pods with seeds of legumes than in the other species (Figs 1 and 4). The concentration of Ca was higher in the leaves of komatsuna, bok-choy, and nabana, and lower in the fruit of Solanaceae species and the seed in green pea (Fig 1). The lower accumulation of Ca in fruit and seeds could be due to the distribution of Ca within the plant largely depending on xylem transfer [17], whereas fruit and seeds mainly use phloem transfer to accumulate minerals [18]. The large difference in Ca concentration between leaf and fruit (Figs 1 and 4) supports this prediction.

In general, there was little variation in the concentration of macroelements between different cultivars of each crop species. However, there tended to be a higher level of variation in the concentration of essential microelements in the edible parts of different crops and, in some cases, cultivars (Figs 1, 2, 4 and 5). For example, garland chrysanthemum and Chinese chive leaves contained a higher concentration of Fe, and a larger variation in Fe concentrations was observed between cultivars of lettuce (leaves), tomato (fruit), and eggplant (fruit) (Figs 1 and 4). A large variation was also observed in the concentration of the following microelements in the edible parts of different cultivars: Mn in lettuce, B in broccoli, Cu in cucumber, Mo in radish, turnip, and napa cabbage, and Ni in tomato rootstock and Chinese chive (Figs 1, 2, 4 and 5). The concentration of Zn was higher in the leaves of komatsuna and bok-choy, and lower in the fruits of Solanaceae species, with little variation between different cultivars of each species (Figs 1 and 4).

There also tended to be higher variation in the concentration of nonessential elements than essential macroelements, with greater levels of variation between cultivars in some cases (Figs 2, 3, 5 and 6). For example, the leaves of Chinese chive showed a remarkably higher concentration of V, Cr, and Ba as well as the essential microelements Fe and Ni (Figs 1–3).

Among the elements analyzed in this study, Fe, Zn, Cu, Ca, Mg, and Se are considered to be particularly important elements for biofortification [5]. Therefore, the information obtained from this study will be of use for selecting or breeding biofortified crops or cultivars. For example, we found that even within the genus *Brassica*, the average concentration and/or the level of variation in the concentration of each of these elements in the leaves differed between species, varieties, or cultivars (Figs 1 and 2).

From a food safety perspective, Cd, As, Cs, and Sr pose a particularly important risk to human health [19, 20]. We found that the leaves of some cultivars of garland chrysanthemum and lettuce exhibited higher concentrations of Cd (Fig 3). It has previously been shown that Cd is homologous to Zn, with the two elements exhibiting similar behaviors during uptake and transport in some plant species [21, 22]. However, although the leaves of garland chrysanthemum contained a higher concentration of Zn as well as Cd, all cultivars of lettuce had lower concentrations of Zn than other those of species (Fig 1), suggesting that the specificity of the uptake and transport systems for Cd and Zn varies even within the same family. Among the nonleaf vegetables, the roots of carrot and the fruit of eggplant contained a higher concentration of Cd, with little variation between the cultivars (Fig 6). It has previously been reported that eggplant often accumulates high concentrations of Cd in its fruit due to the effective xylem loading of this element [23].

Cucumber and eggplant fruits tended to contain higher concentrations of As (Fig 6). Interestingly, variations in the concentration of Sr among different crop species and cultivars were almost equal to variations in Ca, while the concentrations of Mg and Ba showed different trends (Figs 1, 3, 4 and 6). Sr is homologous to Ca, and it has previously been reported that the concentrations of these two elements are highly correlated in the leaves of 138 plant families growing under different environmental conditions [12]. Therefore, this finding implies that we must be careful when cultivating crops that have been selected or bred for Ca-biofortification in areas that are contaminated with radioactive Sr.

There was a high level of variation in the concentration of Cs in the edible parts of different species and cultivars, with a lower concentration tending to be found in fruits or seeds (Figs 3 and 6). The leaves of bok-choy showed the highest average concentration of Cs, but there was also considerable variation between cultivars. The variation in the concentration of Cs that was observed between different crop species was not consistent with that seen for K and Na, which are homologous elements, but was similar to that of Rb (Figs 1–6).

### Ionome of the leaves

It is generally difficult to examine phylogenetic influences on plant ionomes because they are affected by the growth environment, such as the soil nutrient status [2]. However, in the present study, we analyzed the ionome of the leaves of various crop species, including tomato, grape tomato, eggplant, and their rootstocks, which were cultivated under the same field conditions, allowing us to compare these in the absence of any environmental effects.

**Fig 7:**
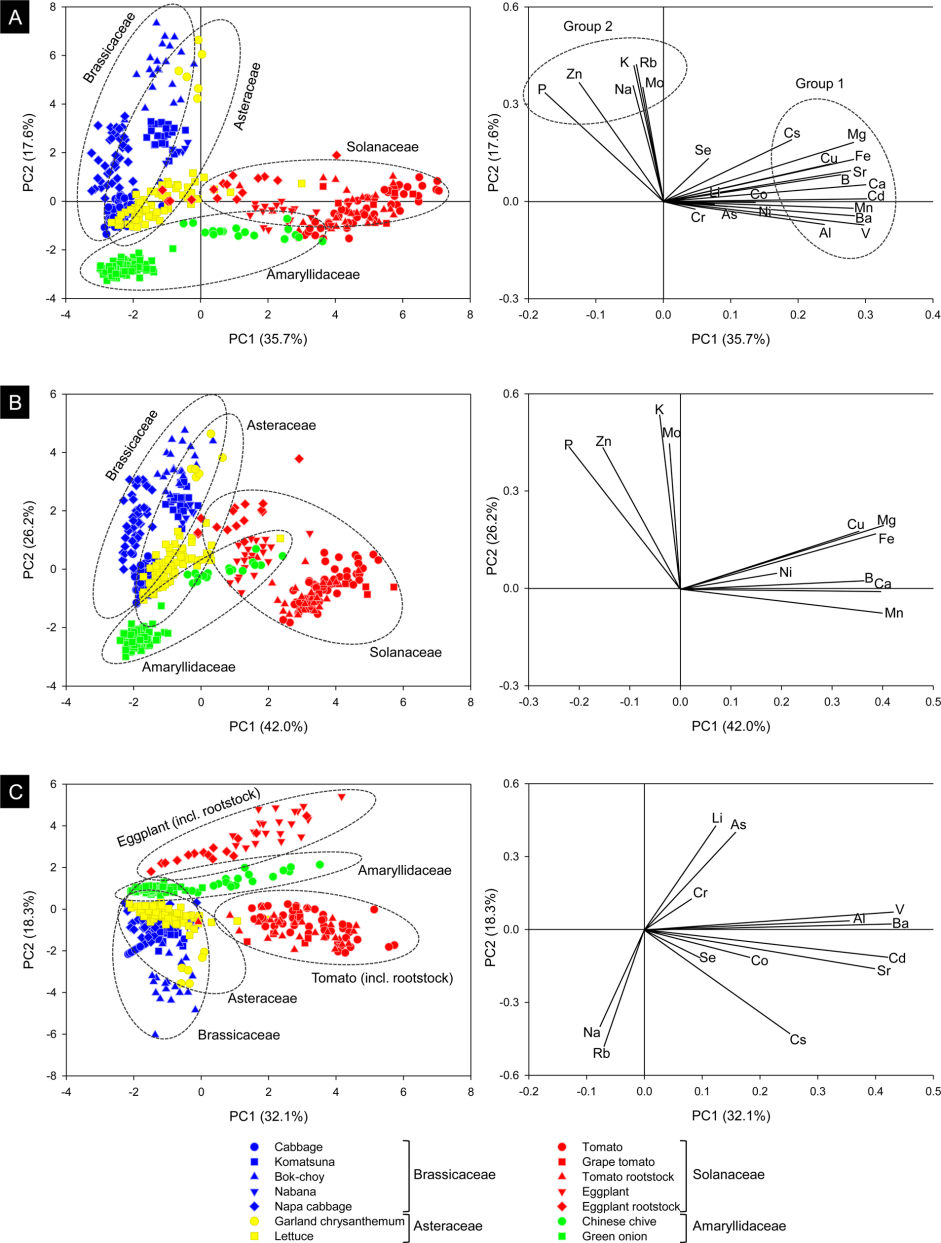
Principal component analysis (PCA) of elements in the leaves of plants analyzed in this study. A: Scores on PC1 and PC2 with all elements included and the corresponding loading plot. B: Scores on PC1 and PC2 with only the essential elements included and the corresponding loading plot. C: Scores on PC1 and PC2 with only the nonessential elements included and the corresponding loading plot.

First, we compared the ionome of the leaves among species using principal component analysis (PCA), including the concentration of all elements (Fig 7A), essential elements (Fig 7B), or nonessential elements (Fig 7C) as the variable. The PCA scores were then presented on the basis of combinations of PC1 and PC2. When conducting the PCA with all elements, the score plots were loosely grouped according to family, although lettuce and garland chrysanthemum (both in Asteraceae) were plotted separately along PC2 (Fig 7A). Brassicaceae showed smaller variations in PC1 and larger variations in PC2, whereas Solanaceae and Amaryllidaceae exhibited larger variations in PC1 and smaller variations in PC2. In the loading plot, elements were loosely classified into two groups; groups 1 and 2 mainly contributed to PC1 and PC2, respectively (Fig 7B). Interestingly, Zn was plotted in group 2, separately from its homologous elements in group 1, and showed similar direction to P. The interaction between Zn and P in their uptake and transport [1] may be related to this result. The results of the PCA that only included the essential elements showed nearly the same trends as the results of the PCA that included all the elements (Fig 7C). In contrast, in the PCA that only included the nonessential elements, tomato and eggplant (both including rootstock) were separated by PC2 (Fig 7C), indicating that they differ in the uptake and/or translocation of nonessential elements despite belonging to the same family.

**Fig 8:**
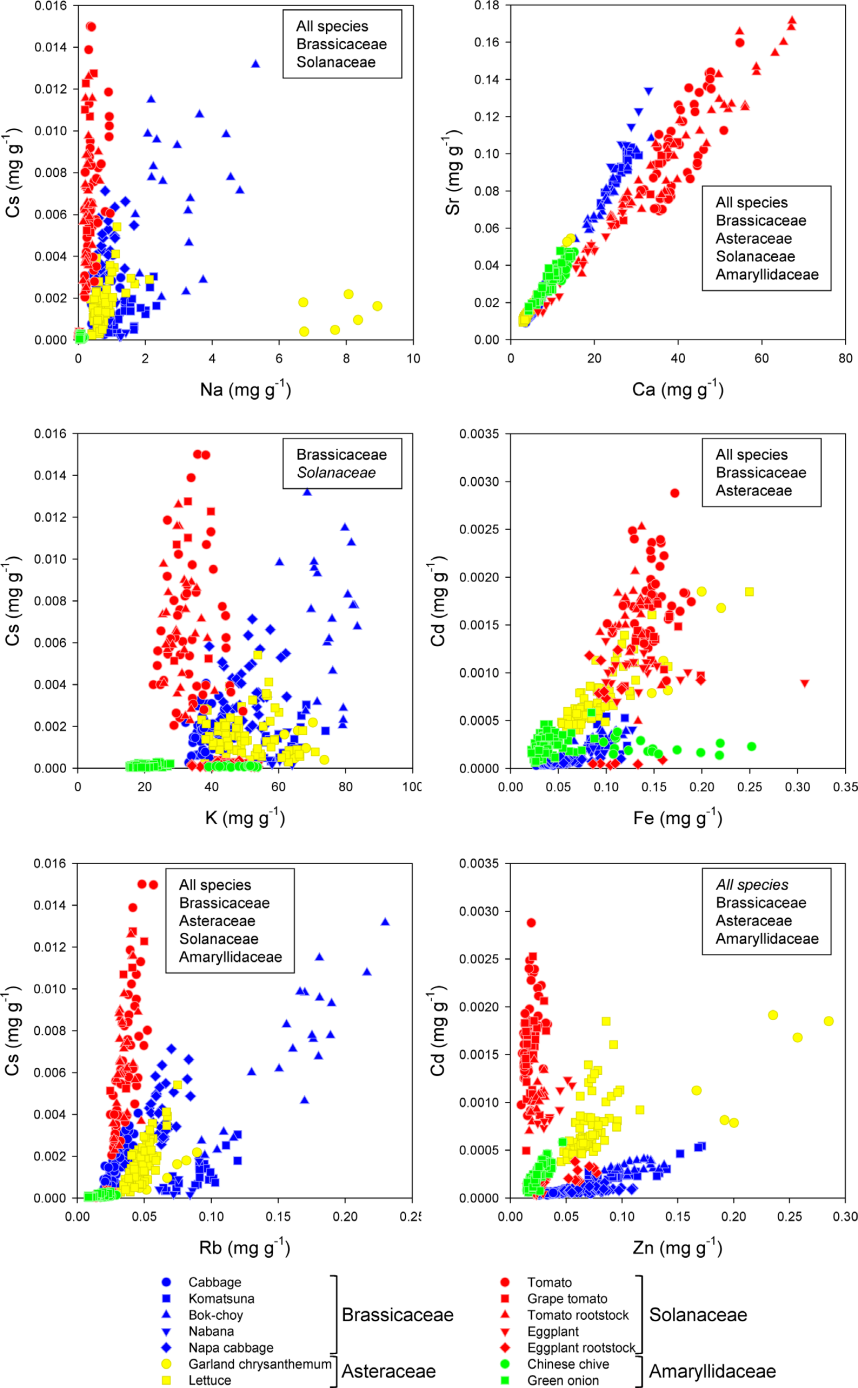
Correlation between the concentrations of pairs of homologous elements that possibly share the same uptake/transport system. Families with a significant correlation (p < 0.01) are shown in a box on each graph (italics = negative correlation).

We then examined the correlation between the concentration of each pair of elements (Cs-Na, Cs-K, Cs-Rb, Sr-Ca, Cd-Fe, and Cd-Zn), which have been reported to partly share the same uptake/transport systems in plants [12, 24–27]. The concentration of Cs was only significantly correlated with that of Na in Brassicaceae, and Cs and K were only significantly correlated in Solanaceae (Fig 8). In contrast, a significant correlation between Cs and Rb was observed in all plant families, although the slope of this relationship differed between families. This suggests that these two elements at least partly share the same uptake/transport systems, but their affinities differ between plant families. Sr was highly correlated with Ca, which may be due to their similar behavior in both plants and the soil [28], and corresponds to the earlier findings for the edible parts (Figs 1, 3, 4 and 6). However, there were slight differences in the slope of this relationship between Solanaceae and other families (Fig 8), implying that the uptake/transport systems in Solanaceae have a slightly higher affinity for Ca.

The concentration of Cd was significantly correlated with the concentration of Fe in Brassicaceae and Asteraceae, and Zn in both these families plus Amaryllidaceae (Fig 8). The slope of this relationship differed greatly between families, however. The concentration of Cd was not significantly correlated with the concentrations of Fe and Zn in Solanaceae, which is particularly interesting since the accumulation of Cd in eggplant fruit often poses a risk to human health. It has been suggested that the xylem-loading citrate transporter gene *FRD3* is responsible for the transport of Cd from the roots to the shoots in *Solanum* spp. [29] and is involved in loading citrate into the xylem, ensuring the delivery of Fe from the roots to the shoots [30]. Therefore, the lack of a significant correlation between Cd and Fe concentrations in the leaves of Solanaceae suggests that Cd uptake and transport mechanisms are very complicated in this family.

### Ionomic interactions between the leaves and fruit

We examined the correlation between the concentrations of each element in the leaves and fruit of tomato and eggplant (Table 2). Correlation coefficient was calculated using the average concentration in each cultivar. The relationship between these was found to differ between these two species, despite both belonging to family Solanaceae. In eggplant, there was no significant correlation between the leaves and the fruit for any element. In contrast, in tomato, there was a significant positive correlation between the concentration of several elements in the leaves and the fruit, particularly for nonessential elements (Table 2). Although the fact that many elements showed no such relationship suggests that it would be difficult to predict the fruit ionome based on the leaf ionome, re-examination with sufficient sample size is necessary in the future study.

**Table 2:** Correlation coefficients and ratio of the concentrations of each element in the leaves and fruit of different cultivars of tomato and eggplant.

		Correlation coefficient		Fruit/leaf ratio (±SE)	
		Tomato (n = 16)	Eggplant (n = 7)	Tomato (n = 16)	Eggplant (n = 7)
Essential	K	0.478	−0.331	1.34 ± 0.05	0.87 ± 0.03
	P	0.456	−0.431	1.88 ± 0.07	1.32 ± 0.10
	Ca	0.521*	0.451	0.02 ± 0.00	0.07 ± 0.00
	Mg	0.429	0.104	0.43 ± 0.02	0.66 ± 0.02
	Fe	0.495	−0.157	0.41 ± 0.04	0.30 ± 0.03
	Mn	0.119	0.685^†^	0.08 ± 0.00	0.27 ± 0.01
	B	0.373	−0.016	0.25 ± 0.01	1.31 ± 0.08
	Zn	0.243	0.491	1.28 ± 0.07	1.07 ± 0.04
	Cu	0.429	0.401	0.67 ± 0.02	0.54 ± 0.05
	Mo	0.787**	0.300	0.71 ± 0.04	1.03 ± 0.11
	Ni	0.611*	−0.081	0.47 ± 0.02	0.41 ± 0.03
Nonessential	Li	0.502*	0.146	0.16 ± 0.01	0.04 ± 0.01
	Na	0.600*	0.570	0.49 ± 0.03	1.55 ± 0.12
	Al	0.443	−0.252	0.30 ± 0.04	0.76 ± 0.11
	V	0.347	0.410	0.02 ± 0.01	0.02 ± 0.01
	Cr	0.608*	−0.386	1.26 ± 0.04	0.92 ± 0.03
	Co	0.100	0.593	0.42 ± 0.03	0.41 ± 0.03
	As	0.673**	−0.473	0.41 ± 0.22	0.12 ± 0.01
	Se	−0.019	0.503	0.72 ± 0.11	0.43 ± 0.09
	Rb	0.557*	0.730^†^	0.66 ± 0.02	0.90 ± 0.04
	Sr	0.176	0.439	0.01 ± 0.00	0.05 ± 0.00
	Cd	0.401	0.700^†^	0.15 ± 0.01	0.57 ± 0.02
	Cs	0.679**	0.661	0.14 ± 0.01	0.22 ± 0.02
	Ba	0.329	0.034	0.01 ± 0.00	0.06 ± 0.01

^†^(only for eggplant), *, and ** indicate statistical significance at *P* < 0.1, 0.05, and 0.01, respectively.

Although the transport and distribution of inorganic elements are mediated by both the xylem and phloem transportation systems, different elements have different levels of dependency on each of these [17]. In the phloem, K, Mg, P, and Na usually have a high mobility, Fe, Zn, Cu, B, and Mo have an intermediate mobility, and Ca and Mn have a low mobility [1]. The fruit/leaf ratio for the concentration of each element may reflect its mobility in the phloem and the efficiency of xylem transport of the element into the fruit (Table 2). The trends in these ratios were nearly the same in tomato and eggplant, with the exception of Ca, Mn, B, Na, and Cd, which had a higher ratio in eggplant than tomato, implying more efficient transport in the phloem and/or higher contribution of xylem transport [23] of these elements resulting in their higher accumulation in the fruit of this species (Figs 1–6).

## Conclusion

This study applied ionomics to various vegetable species and cultivars that were cultivated under nearly identical conditions in large agricultural fields, allowing differences in the concentration of various essential and nonessential elements in the edible parts of crops to be compared with minimal environmental effects. This provides new insights into phylogenetic influences on the ionome which will aid our understanding of the mineral uptake and transport mechanisms in plants and may contribute to improving human health.

## Supporting Information

S1 Table Chemical properties of the field soils used in this study. (PDF)

S2 Table Average mineral concentration (mg g^-1^) of each element in leaves of Komatsuna4 in 2011 and 2012. (PDF)

S3 Table Mineral concentration (mg g^-1^) in edible or nonedible part of each cultivar. (XLSX)
