# Supplementary material for: The Ionomic Study of Vegetable Crops
Source: PLoS One. 2016 Aug 1;11(8):e0160273. doi: 10.1371/journal.pone.0160273 (PMC4968822; doi:10.1371/journal.pone.0160273)
Supplement: S1 Table — (PDF) [file pone.0160273.s001.pdf]

S1 Table. Chemical properties of the field soils used in this study[a].

K (µg g^{-1})	Truog P (µg g^{-1})	Ca (µg g^{-1})	Mg (µg g^{-1})	Fe (µg g^{-1})	Mn (µg g^{-1})	B	Zn (µg g^{-1})	Cu (µg g^{-1})	Mo (ng g^{-1})	Ni (ng g^{-1})	Li (ng g^{-1})	Na (µg g^{-1})
362 ±9	181 ±10	2153 ±60	225 ±5	2.78 ±0.33	10.0 ±1.1	ND[b]	0.796 ±0.115	27.6 ±7.5	11.0 ±0.4	85.4 ±4.4	34.8 ±2.0	23.4 ±0.9

Al (µg g^{-1})	V (ng g^{-1})	Cr (ng g^{-1})	Co (ng g^{-1})	As (ng g^{-1})	Se (ng g^{-1})	Rb	Sr (µg g^{-1})	Cd (ng g^{-1})	Cs (µg g^{-1})	Ba (µg g^{-1})	pH (H_2O)
2.89 ±0.23	3.55 ±0.21	224 ±8	17.4 ±1.3	30.2 ±0.8	5.12 ±0.70	ND	9.15 ±0.17	33.7 ±1.2	0.496 ±0.062	17.7 ±0.3	5.43 ±0.02

[a]Concentration of elements except for P were determined by ammonium acetate extraction. Available P concentration was determined by Truog method. Soil pH was determined by using a 1 : 2.5 soil to water ratio using pH meter. See Watanabe et al. (2015) for details.

[b]Not determined
