# Supplementary material for: The Ionomic Study of Vegetable Crops
Source: PLoS One. 2016 Aug 1;11(8):e0160273. doi: 10.1371/journal.pone.0160273 (PMC4968822; doi:10.1371/journal.pone.0160273)
Supplement: S2 Table — (PDF) [file pone.0160273.s002.pdf]

S2 Table. Average mineral concentration (mg g^{-1}) of each element in leaves of Komatsuna4 in 2011 and 2012.

	K	P	Ca	Mg	Fe	Mn	B	Zn	Cu	Ni	Na	Al	Cd	Sr
2011	58.4	6.35	28.2	3.48	0.107	0.0380	0.0197	0.110	0.00648	0.00101	2.00	0.0450	0.000240	0.0992
2012	53.8	7.05	24.1	3.35	0.132	0.0382	0.0241	0.114	0.00663	0.00117	2.09	0.0306	0.000504	0.0805
